# Dr. Jacob F. Raub

**Published:** 1906-07

**Authors:** 


Dr. Jacob F. Raub, of Wahington, D. C., died at the U. S. Pension Office, on the afternoon of May 21, 1906, aged 66 years. His health had been somewhat infirm for several months but he kept on duty the greater part of the time as one of the principle examiners, and on the day of his death went to the office as usual. In the late afternoon he felt some precordial oppression and reclined upon a sofa. Before he could be removed to his home he expired, his death being due to disease of the heart. Dr. Raub served as Medical Referee of the pension office from 1897 to 1902 and was regarded as a capable officer, enjoying the respect of his superiors and associates in the service. His military services were remarkable. He enlisted as a private soldier in the 129th regiment Pa. Vols., in 1862; in 1864 he was appointed assistant surgeon of U. S. Vols. and was assigned to duty in Washington; at his own request he was sent to the field, being assigned to duty with the Sixth Army Corps, serving in the campaign from the Rapidan to Petersburg; later he was transferred to the Fifth Army Corps where he served as one of the staff of operating surgeons. For gallantry at Hatcher's Run, February 5, 1865, he was awarded a congressional medal of honor. He was a member of the Guard of Honor around the body of President McKinley at the White House in 1901.

Dr. Raub is survived by his wife and a married daughter who live in Washington, and by a son, Austin J. Raub, of Buffalo. His remains were buried at Arlington, the services being in charge of the Grand Army of the Republic.

